# Towards standardising retinal OCT angiography image analysis with open-source toolbox OCTAVA

**DOI:** 10.1038/s41598-024-53501-6

**Published:** 2024-03-12

**Authors:** Gavrielle R. Untracht, Madeleine S. Durkee, Mei Zhao, Andrew Kwok-Cheung Lam, Bartosz L. Sikorski, Marinko V. Sarunic, Peter E. Andersen, David D. Sampson, Fred K. Chen, Danuta M. Sampson

**Affiliations:** 1 Department of Health Technology, Technical University of Denmark, 2800 Kongens Lyngby, Denmark; 2 School of Biosciences, The University of Surrey, Guildford, GU27XH UK; 3 Department of Radiology, University of Chicago, Chicago, IL 60637 USA; 4 Centre for Myopia Research, School of Optometry, Faculty of Health and Social Science, The Hong Kong Polytechnic University, Hung Hom, Kowloon, Hong Kong China; 5 Department of Ophthalmology, Nicolaus Copernicus University, 85-090 Bydgoszcz, Poland; 6 International Center for Translational Eye Research (ICTER), Institute of Physical Chemistry, Polish Academy of Sciences, Kasprzaka 44/52, 01-224 Warsaw, Poland; 7 Department of Medical Physics and Biomedical Engineering, University College London, London, WC1E6BT UK; 8 Institute of Ophthalmology, University College London, London, EC1V2PD UK; 9 School of Computer Science and Electronic Engineering, The University of Surrey, Guildford, GU27XH UK; 10 Centre for Ophthalmology and Visual Science (Incorporating Lions Eye Institute), The University of Western Australia, Perth, WA 6009 Australia; 11 Department of Ophthalmology, Royal Perth Hospital, Perth, WA 6000 Australia; 12 Ophthalmology, Department of Surgery, University of Melbourne, Melbourne, VIC 3002 Australia; 13 Department of Optometry, School of Allied Health, The University of Western Australia, Perth, WA 6009 Australia

**Keywords:** Diagnostic markers, Imaging and sensing, Biophotonics, Blood flow

## Abstract

Quantitative assessment of retinal microvasculature in optical coherence tomography angiography (OCTA) images is important for studying, diagnosing, monitoring, and guiding the treatment of ocular and systemic diseases. However, the OCTA user community lacks universal and transparent image analysis tools that can be applied to images from a range of OCTA instruments and provide reliable and consistent microvascular metrics from diverse datasets. We present a retinal extension to the OCTA Vascular Analyser (OCTAVA) that addresses the challenges of providing robust, easy-to-use, and transparent analysis of retinal OCTA images. OCTAVA is a user-friendly, open-source toolbox that can analyse retinal OCTA images from various instruments. The toolbox delivers seven microvascular metrics for the whole image or subregions and six metrics characterising the foveal avascular zone. We validate OCTAVA using images collected by four commercial OCTA instruments demonstrating robust performance across datasets from different instruments acquired at different sites from different study cohorts. We show that OCTAVA delivers values for retinal microvascular metrics comparable to the literature and reduces their variation between studies compared to their commercial equivalents. By making OCTAVA publicly available, we aim to expand standardised research and thereby improve the reproducibility of quantitative analysis of retinal microvascular imaging. Such improvements will help to better identify more reliable and sensitive biomarkers of ocular and systemic diseases.

## Introduction

The imaging and assessment of retinal microvasculature are important for studying, diagnosing, monitoring, and guiding the treatment of ocular and systemic conditions^1^. Optical coherence tomography angiography (OCTA) is a commercially available imaging technique that enables the visualisation and characterisation of the retinal and other microvascular networks. Retinal OCTA is attractive to clinicians as it is fast, non-invasive, does not require the administration of dye, and can provide images of the microvascular network from different retinal depths without obscuration by dye leakage^2^. OCTA is becoming a popular imaging tool in ophthalmic and optometric care and the field continues to grow rapidly^3^. Major challenges include a lack of standardised data collection and transparent analysis methods and the absence of standardised image grading, interpretation methods, and metrics^2,4–6^. Open data, software sharing, and cross-comparison and pooling of data from different studies remain uncommon in publications. The lack of good open-science practice has impeded the building of large databases of annotated OCTA images of healthy and diseased retinas that are necessary to study and define the characteristics of specific conditions^3^. Fortunately, there is growing interest within the research community in delivering tools that enable the visualisation and quantification of microvasculature. Several tools originally developed for microscopy can be applied to OCTA images^7–10^. A few toolboxes and image analysis workflows have also been developed specifically for OCTA^11–16^. However, due to the limited number of metrics they deliver or their complexity, and/or the requirement for user intervention, they can be challenging for daily use by eye healthcare professionals. So far, there is no consensus on a standard processing framework for OCTA^6^.

Recently, we introduced a user-friendly, open-source toolbox, OCTAVA (OCTA Vascular Analyser), to automate the pre-processing, segmentation, and quantitative analysis of *en face* OCTA maximum intensity projection images of microvasculature^17^. We demonstrated the utility of OCTAVA for assessing differences in OCTA-derived cutaneous microvascular metrics in people with type 2 diabetes^18^. Here, we present an enhanced version of OCTAVA for comprehensive characterisation of the retinal microvascular network and foveal avascular zone (FAZ). OCTAVA delivers seven microvascular metrics, including vessel area density (VAD), vessel length density (VLD), total, mean, and median vessel length (VL), mean and median vessel diameter, branchpoint density (BD), tortuosity, and fractal dimension (FD) for the whole image. For retinal OCTA, we added the capability to assess metrics for subregions that are either user-defined, defined by the Early Treatment Diabetic Retinopathy Study (ETDRS) grid, or a grid of nine equal squares. We also introduced the FAZ metrics: FAZ area, perimeter, circularity, acircularity index, axis ratio, and vessel density within a 300-µm width ring surrounding the FAZ (FD-300). The user inputs the size of each image to ensure they are corrected for transverse magnification errors related to differences in the eye’s axial length between individuals^19^. OCTAVA has two different operating modes: one to process individual images and a batch processing mode to analyse multiple images without additional user input. In both cases, the same image processing workflow is utilised. The toolbox saves binarized, skeletonized, and FAZ-segmented images—so the user can visually inspect the performance of the analysis. The quantitative analysis results are saved to an Excel file for easy access to raw data for further analysis. Access to images generated at different stages of the image analysis workflow and numerical data saved in an easily accessible format maximise transparency and shareability between users.

In the following, we validate the performance of OCTAVA on retinal images obtained using four OCTA commercial instruments: Cirrus 5000 (Carl Zeiss Meditec AG, Germany), Revo NX 130 (Optopol Technology Sp. z o.o., Poland), RTVue-XR Avanti (Optovue Inc., USA), and Spectralis OCT2 module (Heidelberg Engineering GmbH, Germany), each imaging different healthy study participant cohorts. We establish OCTAVA parameters recommended for use with these four instruments when retinal OCTA images are analysed. We demonstrate that the toolbox can determine metrics for characterising the retinal microvasculature independent of the instrument employed. We compare metrics generated by OCTAVA with those generated by commercial in-built software packages (when available) and show that using OCTAVA leads to less variance in OCTA-derived retinal microvascular metrics generated using different instruments and different study cohorts. Our toolbox is available in open repositories; the source code is available on Github^20^, and a compiled MATLAB app or standalone software version—for a user without a MATLAB license—is available on Sourceforge^21^ or on request. Our intention is to enable retinal OCTA image analysis in a transparent (i.e., with all processing steps visible and clearly explained), and ultimately standardised way. Wide adoption of OCTAVA could enable further optimisation of the toolbox on large datasets of healthy individuals and those with retinal diseases from different instruments acquired at different sites. Additionally, widespread use of a standardised toolbox like OCTAVA will minimise systematic variability in OCTA-derived metrics due to the differences in image analysis software used by different study sites. Ultimately, this will help to generate more reliable microvascular metrics and to build multi-site large datasets, which are necessary to identify the most general and sensitive biomarkers of various health conditions.

## Methods

### OCTA datasets

The characteristics of OCTA instruments and datasets of healthy individuals used for the advancement and validation of OCTAVA are summarised in Table 1. All images derive from retrospective cross-sectional studies undertaken in three academic centres in Australia, Hong Kong (China), and Poland. The data collected in Australia include two scans acquired on the right eye during one imaging session to measure intrasession repeatability. The study protocol from Australia was reviewed by the Office of Research Enterprise and the institutional review board (IRB) of the University of Western Australia (RA/4/20/4275) comprising use of the images from RTVue XR Avanti and the cross-sectional analysis, and it was considered exempt from full ethics review by the board as this was a retrospective review of anonymised imaging and clinical data collected as part of routine clinical care. OCTA images from the Cirrus 5000 and Spectralis OCT2 module were retrieved from two studies approved by the institutional review board (IRB) of The Hong Kong Polytechnic University (reference numbers HSEARS20170414001 and HSEARS20210122008, respectively). The study was exempted from the further approval by the IRB as this was a retrospective review of anonymised images and clinical data collected. OCTA images from the Revo NX 130 were retrieved from a study approved by the Committee of the Collegium Medicum, Nicolaus Copernicus University (reference number KB 898/2018) and exempted from further approvals. All studies followed the tenets of the Declaration of Helsinki and informed consent was obtained from all subjects.

**Table 1:** Characteristics of the commercial OCTA instruments and datasets.

Instrument, manufacturer	Cirrus 5000, Carl Zeiss Meditec AG^24^	Revo NX 130, Optopol Technology^25^	RTVue-XR Avanti, Optovue Inc.^26^*	Spectralis OCT2 module, Heidelberg Engineering^27^
Data collection city, country	Hong Kong, China	Bydgoszcz, Poland	Perth, Australia	Hong Kong, China
Image quantity/number of participants	32	30	31	27
Instrument definition of analysed retinal layer	SRLS	SVP	SVP, total retina**	SVC
Defined retinal layer segmentation upper boundary	ILM	ILM	ILM (SVP) ILM (total retina)	ILM
Defined retinal layer segmentation lower boundary	ILM + 70% $\times$ T_ILM-OPL_	IPL (offset: -15 µm)	IPL (offset: -10 µm) (SVP) OPL (offset: +10 µm) (total retina)	IPL (offset: -17 µm)
Image size [mm]	3 $\times$ 3	3 $\times$ 3	3 $\times$ 3	3 $\times$ 3
Number of A-scans along *x*-axis	245	320	304	512
Number of B-scans along *y*-axis	245	320	304	512
Central wavelength [nm]	840	850	840	880
Imaging speed [A-scan rate, Hz]	68,000	130,000	70,000	85,000
Axial resolution [µm]***	5	5	5	5.7
Transverse resolution [µm]	15	18	22	11.4
Sampling density [1]****	1.23	1.92	2.23	1.95
Projection artefacts removal function	Yes	No	Yes	Yes
Angiogram construction algorithm	Optical micro-angiography (OMAG)^28^	Spectral domain absolute complex difference^29^	Split-spectrum amplitude-decorrelation (SSADA)^30^	Full-spectrum amplitude decorrelation^31^
En face projection	Maximum intensity projection	Average intensity projection	Maximum intensity projection	Sum projection + contrast function
Automated image quality indicator	Yes	Yes	Yes	Yes
Instrument name of the quality indicator	Zeiss signal strength	Quality index	Scan quality index	Q score
Image quality indicator threshold (range)	> 6 (0–10)	> 6 (0–10)	≥ 6 (0–10)	> 15 (0–40)
Interscan time [ms]	Not specified	Not specified	Not specified	Not specified
Maximum detectable blood flow velocity [mm/s]	Not specified	Not specified	Not specified	Not specified
Instrument defined ocular axial length [mm]*****	24.46	22.10	23.95	24.39
*En face* image exported from the OCTA instrument: number of pixels and image type	717 $\times$ 717 tif	640 $\times$ 640 bmp	304 $\times$ 304 png	1000 $\times$ 1000 bmp

*SRLS* superficial retinal layer slab, *SVP* superficial vascular plexus, *SVC* superficial vascular complex, *ILM* internal limiting membrane segmented layer, *T*_*ILM-OPL*_ thickness between ILM and the outer plexiform layer (OPL), *IPL* inner plexiform layer.

*Two sets of data for each participant acquired in one imaging session were used to calculate intrasession repeatability for metrics generated by OCTAVA and in-built software.

**OCTA image of the total retina was used only for foveal avascular zone analysis.

***Resolution is measured in tissue.

****Sampling density is defined as transverse resolution divided by scan separation, which is the transverse scan length divided by the number of samples (either number of A-scans along the *x*-axis or B-scans along the *y*-axis.

*****Corrected for transverse image magnification error using the Littmann-Bennett formula as per Ref.^19^.

Information about the instrument-defined axial length from personal correspondence (Revo NX 130 and RTVue-XR Avanti) and^32^ (Cirrus 5000 and Spectralis OCT2) Unit of 1 indicates dimensionless.

*En face* OCTA maps used in the study were generated using the default automated segmentation boundaries generated by the in-built commercial software. In most cases, the *en face* OCT maps comprise only the superficial vascular plexus, named and defined slightly differently between instruments. For the RTVue-XR, an *en face* map comprising the whole retinal thickness is only used for the FAZ segmentation. Only good-quality OCTA images were included for analysis. All OCTA images were pre-screened to exclude images with significant motion artefacts, projection artefacts, images with low signal strength, distortion, out of focus, and lack of centration at the foveal avascular zone. To do so, we applied a combination of automated image quality indicators available in the OCT data acquisition software and visual inspection^22,23^.

### OCTAVA software

**Figure 1:**
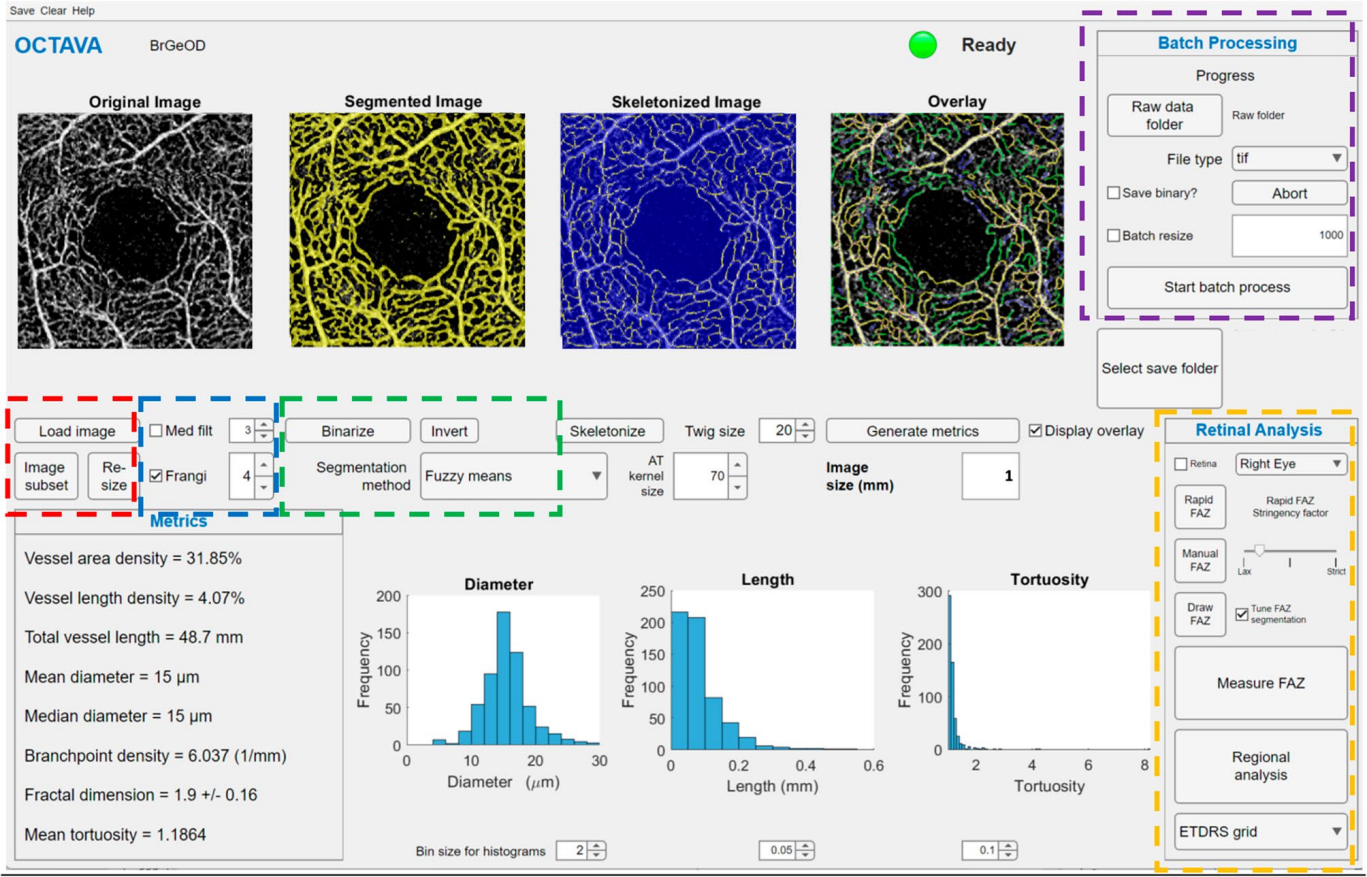
Updated OCTAVA graphical user interface with added metrics for retinal analysis. Coloured boxes indicate user controls for optimising image processing. Red box: the user can modify the image by down-sampling or selecting a subregion of the full image for faster processing or by up-sampling to improve the accuracy of the metrics. Blue box: median filter and Frangi filter for improving segmentation. Green box: choice of the segmentation algorithm. Purple box: controls for batch processing. Yellow box: controls for FAZ segmentation and regional analysis.

OCTAVA enables the processing and quantitative analysis/characterisation of OCTA maximum intensity projection (MIP) images downloaded from the OCTA instrument directly and saved in an open-source format, such as jpg, png or tif. The current version of the toolbox is fully developed in MATLAB. The previous version^17^ utilised the ImageJ-MATLAB package to access ImageJ libraries. The functions that required this ImageJ interface have been replaced with MATLAB algorithms to enable faster image processing and analysis. The graphical user interface (GUI) has also been updated to include the newly implemented features and additional metrics that are required for the assessment of retinal microvasculature (Fig. 1). Because our software is open source and developed in MATLAB, it can easily be modified to adapt to the needs of the research and clinical community while still being easy to use without the need for modification of the back-end code. A MATLAB license is required to modify the software; however, a stand-alone version of OCTAVA can be used without local access to MATLAB. This standalone version is compatible with PC, Mac, and Linux operating systems.

OCTAVA has two different operating modes: the default mode, which allows the user to process individual images with the aim of optimising the analysis protocol for specific image types, and a batch-processing mode, which enables the user to analyse multiple images without additional user input (purple box in Fig. 1). In both cases, the same image processing workflow is utilised; each of these steps is described in detail in^17^ and summarised briefly below. Additionally, a new feature has been added to the batch-processing mode to resize all images to the same number of pixels. This feature is important to ensure that kernel-based filters, such as the Frangi and median filters, are applied consistently across all images regardless of the instrument used for data acquisition. FAZ segmentation is also a new function in OCTAVA.

A wide range of metrics has been included in OCTAVA to maximise the flexibility to identify the most powerful biomarkers. In addition to the general/global microvascular metrics applicable to any OCTA MIP image^17^, the user has the option of generating metrics within pre-defined regions (subregions defined by the defined by the user, the ETDRS grid, or a grid of nine equal squares) to facilitate comparison with other retinal OCTA instrumentation and software (Suppl. Fig. S1). In OCTAVA, the ETDRS grid is centred on the centroid of the FAZ identified after FAZ segmentation, and the grid of squares is centred on the image centre.

### OCTAVA image processing workflow

An overview of the OCTAVA image processing workflow is shown in Suppl. Fig. S2. The image quality of OCTA MIPs is first manually assessed by the user to determine if the image quality is sufficient to proceed. Images should have few motion artefacts relative to the number of vessels and should have sufficient signal strength that the vascular network is generally visible. To reduce noise in the image, the user can opt to use a median filter, with variable kernel size. Then a 2D Frangi “vesselness” filter is applied since the objects of interest are blood vessels. The Frangi filter is commonly used in analysing angiography images since it reduces the impact of intensity variations along a vessel and suppresses background noise, thereby improving image segmentation. The Frangi filter is iterative and applies kernels up to a maximum size; the maximum kernel size of the Frangi filter can be changed by the user to enable optimisation. Next, the pre-processed image is segmented into two regions representing “vessels” and “not vessels”. Two segmentation methods are available in OCTAVA: fuzzy thresholding^33^ and adaptive thresholding^34^. These methods were selected based on a literature review and experimental analysis of several methods^17^. In most cases, we found that fuzzy thresholding provided the best segmentation. However, we included adaptive thresholding for comparison since it is commonly employed in other retinal OCTA studies.

After segmentation, the binarized image is skeletonized using a MATLAB built-in 3D thinning algorithm^35^, and a heatmap of vessel diameter is generated using a Euclidian distance transform^36^. The connectivity of the network is analysed by converting the skeletonized image into an undirected graph structure, from which the branchpoints are identified, and vessels are classified based on their connectivity to the network^37^. Segments are connected to other vessels at both endpoints, branches are connected to another vessel at one end but have one free endpoint, and isolated elements are not connected to other vessels at either end. Isolated elements and branches below a certain length (specified using the “twig size” control in the OCTAVA GUI) are most likely noise and are excluded from the analysis. Twig size, specified in pixels, may require adjustment based on the presence of noise and pixel density in individual datasets. In a perfect representation of the microvascular network, all vessels would be connected to the network at both endpoints. This is not the case in practice, however, because of multiple factors, including one or more instances of low signal-to-noise ratio, projection artefact, inadequate segmentation (especially with the smaller vessels in the retina), and/or below-threshold flow speed (given the fact that OCTA can only visualise vessels with flow speeds above a certain threshold, dependent on the acquisition speed of the instrument).

In addition to the general/global microvascular metrics applicable to any OCTA MIP image^17^, the user has the option of generating additional retina-specific metrics related to the FAZ. The binarized image is used to generate FAZ segmentation using a two-step process. First, the user initialises the contour of the FAZ with an approximate segmentation. This approximation can be done via one of three methods to ensure optimal segmentation (yellow box in Fig. 1), according to need: automatically based on the segmented image (Rapid FAZ); by manually selecting points to outline the contour (Manual FAZ); or by drawing the contour (Draw FAZ). The three options are available since the Rapid FAZ initialisation and optimisation process sometimes performs poorly for noisy images. The Rapid FAZ segmentation is generated through dilation, gaussian filtering, and thresholding of the binary vessel segmentation. Any extraneous small objects far from the centre of the image are removed. A stringency factor option allows the user to dilate (low stringency) or erode (high stringency) the default rapid segmentation initialisation. The result of this Rapid FAZ segmentation can be accepted as the final segmentation or used to initialise Chan-Vese active contouring^38^ to fine-tune the segmentation. If the rapid approximation is insufficient, the Manual FAZ initialisation can be used to select points near the FAZ boundary as an initialisation for active contouring. Alternatively, the Draw FAZ option allows the user to provide a manual segmentation of the FAZ, if so preferred. The same FAZ segmentation protocol generally works well for all images acquired with the same OCTA instrument. Once the FAZ segmentation protocol has been identified, the settings can be specified on the OCTAVA graphical user interface so that they will be applied to all images if batch processing is used. This process is fully automated unless manual initialisation of the contour is required. An image showing the FAZ segmentation is saved so that the user can retrospectively assess the segmentation and re-process individual images in the unlikely case that the segmentation failed. Finally, metrics are evaluated and written to a spreadsheet for post-processing and statistical analysis.

### Metrics for characterisation of microvascular network and foveal avascular zone

As discussed in detail in other papers^3,7,17^, the complexity of retinal microvascular architecture in health and disease signifies that full characterisation of changes in vessel morphology will require multiple metrics. Based on an extensive qualitative literature review, we chose seven metrics to comprehensively characterise the microvascular network (vessel area density, vessel length density, total, mean, and median vessel length, mean and median vessel diameter, branchpoint density, tortuosity, and fractal dimension for the whole image or a subregion as defined above) and six others to assess the foveal avascular zone (FAZ area, perimeter, circularity, acircularity index, axis ratio, and vessel density within a 300-µm width ring surrounding the FAZ (FD-300)). A description of the metrics used in this study and a brief indication of why they are important is presented in Suppl. Table S1.

### OCTAVA performance assessment

The vessel segmentation algorithms were evaluated in our previous paper^17^ and so this evaluation is not repeated here. Rather, we focus on evaluating the optimum values for OCTAVA parameters for processing retinal OCTA images (MIPs) delivered by the Cirrus 5000, Revo NX 130, RTVue-XR or Spectralis OCT2 module towards establishing standardised procedures that can be applied to all OCTA retinal instruments.

### Frangi filter maximum kernel size for retinal images

To assess the optimal Frangi filter maximum kernel size for our retinal images, 40 images were chosen from our dataset: ten per OCTA instrument. These images were processed using OCTAVA for varying Frangi filter maximum kernel size from 1 to 8 pixels. Image panels of binarizations using different maximum kernel sizes were prepared. Each panel included the original image and eight randomly ordered binarized images to be graded by human readers. All images were up-sampled to 1000 $\times$ 1000 pixels so that the Frangi filter would be applied consistently across datasets from each OCTA instrument. Three graders selected to balance scientific/clinical backgrounds and years of experience in the OCTA field were invited to independently assess the Frangi filtered images (MZ—clinician, four years’ experience; GRU—optical engineer, two years’ experience; MSD—a computer scientist, 6 months’ experience).

The graders were asked to choose the image with the best binarization performance based on visual inspection and comparison with the original image (before binarization). Each grader was given a short tutorial on the grading system reinforced by several illustrative cases. These cases were excluded from the analysed dataset. Graders were blinded to Frangi filter maximum kernel size value, model of the OCTA instrument, and biometric data. In cases of disagreement between two or three graders, the final score for each image (“reference standard”) was established based on the consensus from a real-time discussion between the three graders. DMS, who prepared image panels but did not grade them, moderated the discussion but provided no opinion or influence to affect grader discussions^39^. Inter-grader agreement between the three graders was quantified by the Fleiss kappa coefficient. Cohen’s kappa statistic was used to calculate agreement between each grader and reference standard scores established using SPSS (Version 29.0; IBM, Inc., United States)^40^. This task was undertaken to identify the optimal maximum kernel size for our datasets. Although the maximum kernel size can be chosen differently for each image, identifying a fixed value for the whole dataset is preferred to minimise computational artefacts that cause variations in metric values.

### Twig size value for retinal images

Originally, we planned to apply the same procedure used to establish the maximum kernel size of the Frangi filter to identify the optimal twig size. However, assessing the images proved to be challenging for human readers. The skeletonized images processed with various values of the twig size demonstrate very subtle differences that cannot be detected easily by the naked eye. In lieu of image grading for different twig sizes, we processed 40 images with twig sizes 0, 4, 8, 12, 16, and 20 pixels and examined the change in metric values versus twig size to determine its optimal value.

### Comparison of OCTAVA with in-built OCTA software

We compared metrics delivered by OCTAVA with those delivered by in-built OCTA software packages where such metrics are provided. Prior to this, we determined how many images from each instrument would be needed to demonstrate a statistical difference between OCTAVA and the in-built software packages. The power analysis was based on VAD, which has been the most often studied metric. We used G*Power V.3.1. software^41^ (University of Dusseldorf) to establish that 22 images per instrument would be required to provide a statistical power of 0.8 at alpha = 0.05, assuming a mean value of vessel area density of 40%, difference ± 5% and standard deviation of 3%.

Agreement between the results from OCTAVA and in-built software in commercial OCTA instruments was evaluated using Bland–Altman plots, limits of agreement^42^ and the Spearman correlation coefficient. We also used histogram analysis to examine how OCTAVA-derived and in-built software-derived metrics are distributed. For Bland–Altman analysis, a scatter plot of absolute differences in values between the two software packages (OCTAVA and, in turn, each commercial software) against their means was plotted to confirm that there was no relationship between error and magnitude. If this condition was met, then the bias (mean difference), the standard deviation of the differences (SD of diff.), and confidence limits for the bias (i.e., the limits of agreement, LA, defined as mean difference ± 1.96 SD of the differences) were calculated. The LA represents the range of values for the differences between the methods that can be expected 95% of the time. Paired t-tests were used to examine the significance of the bias. The t-statistic is given by ${t}_{stat}=\overline{d }/SE\left(\overline{d }\right),$ where $\overline{d }$ is the mean difference, and $SE\left(\overline{d }\right)={s}_{d}/\sqrt{n}$ is the standard error of the mean difference calculated under the null hypothesis; ${s}_{d}$—standard deviation and $n$—number of samples. A p-value of 0.01 or less is considered statistically significant. The Spearman correlation coefficient is defined as the covariance of the values acquired with the two software packages divided by the product of the standard deviation of the values acquired with each software. An overview of the metrics available from the in-built software and the corresponding metrics in OCTAVA is presented in Table 2.

**Table 2:** Overview of metrics available in the in-built software packages for different instruments in comparison with equivalent metrics in OCTAVA.

Retinal OCTAVA	Cirrus 5000, Carl Zeiss Meditec AG	Revo NX 130, Optopol Technology	RTVue-XR Avanti, Optovue Inc	Spectralis OCT2 module, Heidelberg Engineering
VAD [%]	Capillary perfusion density [mm^2^/mm^2^]	VAD [%]	Vessel density [%]	N/A
VLD [%]	Vessel density [mm/mm^2^]	Skeleton area density [%]	N/A	N/A
Vessel diameter [μm]	N/A	N/A	N/A	N/A
Vessel length [μm]	N/A	N/A	N/A	N/A
Branchpoint density [nodes/mm]	N/A	N/A	N/A	N/A
Tortuosity [1]	N/A	N/A	N/A	N/A
Fractal dimension [1]	N/A	N/A	N/A	N/A
FAZA [mm^2^]	FAZA [mm^2^]	FAZA [mm^2^]	FAZA [mm^2^]	N/A
FAZ perimeter [mm]	FAZ perimeter [mm]	FAZ perimeter [mm]	FAZ perimeter [mm]	N/A
FAZ axis ratio [%]	N/A	N/A	N/A	N/A
FAZ circularity [1]	FAZ circularity [1]	FAZ circularity [1]	N/A	N/A
FAZ acircularity index [1]	N/A	N/A	FAZ acircularity index [1]	N/A
FD-300 [%]	N/A	N/A	FD-300 [%]	N/A

*N/A *indicates that a metric is not available in that software.

[1] indicates dimensionless.

We now describe instrument-specific aspects of the validation. The Cirrus 5000 in-built software delivers two OCTA metrics to characterise vessel architecture. Vessel density (VLD in OCTAVA) is defined as the total length of perfused vasculature per unit area (mm/mm^2^). Capillary perfusion density (VAD in OCTAVA) is defined as the total area of perfused vasculature per unit area (mm^2^/mm^2^). The analysis is done for the whole image and regions defined by the ETDRS grid. The ETDRS grid, by default, is located at the centre of the image. However, it can be moved and/or re-centred based on the slice navigator position or the centre of the fovea calculated by Cirrus Fovea Finder (an add-on to the main software package that isn’t always available). The ETDRS grid was centred on the fovea for all Cirrus 5000 OCTA images. Cirrus 5000 FAZ analysis tool delivers FAZ area, perimeter, and circularity^24^. The Cirrus 5000, Revo NX 130 (see below) and OCTAVA use slightly different definitions for measuring vessel length density (Table 2 and Suppl. Table S1). To ensure a valid comparison, we rescaled the values of VLD reported by the Cirrus 5000, so as to report and compare total vessel length (VL), as follows. The VLD for the whole image area delivered by the Cirrus 5000 was multiplied by the total image size to obtain a total vessel length in mm (${VLD}_{cirrus}\times (3\times 3) {{\text{mm}}}^{2}).$ The foveal circle VLD was multiplied by the area of a circle of 1 mm diameter $\left({VLD}_{Cirrus}\times \left(\pi \times {0.5}^{2}{\mathrm{ mm}}^{2}\right)\right)$. The VLD density in the parafoveal region was rescaled as ${VLD}_{cirrus}\times \left(\left(\pi \cdot {1.5}^{2} {{\text{mm}}}^{2}\right)-\left(\pi \cdot {0.5}^{2}{ {\text{mm}}}^{2}\right)\right)$. The resulting values were compared with the total vessel length [mm] generated by OCTAVA in the corresponding region.

The Revo NX 130 in-built software delivers VAD defined as the total area of perfused vasculature per unit area in the measurement region (the same as VAD in OCTAVA), skeleton area density (VLD in OCTAVA) defined as the total area of skeletonized vasculature per unit area in a measurement region, and a FAZ analysis tool which delivers FAZ area, perimeter, and circularity. Quantification is available for the whole image and for regions matching the ETDRS grid. The ETDRS grid, by default, is located at the centre of the image. However, it can be moved and/or recentred based on the slice navigator position. The ETDRS grid was centred on the fovea for all Revo NX 130 OCTA images used in this study.

The RTVue-XR in-built software delivers vessel density (VAD in OCTAVA) for the whole image and for subregions defined by the ETDRS grid or in nine squares. The software automatically identifies the foveal centre and the ETDRS grid is centred on the foveal centre. ETDRS grid centration adjustment is also available. The FAZ analysis enables quantification of FAZ area, perimeter, acircularity index and FD-300^26^.

The Spectralis OCT2 module does not have in-built OCTA image analysis software.

We also sought to investigate whether the distribution and variance of metrics acquired by OCTAVA were different from those of the in-built software packages. A Kolmogorov–Smirnov test was used to test the alternative hypothesis that the commercial software packages and OCTAVA produce different distributions of measurements. The alternative hypothesis, that the variances of the computed metrics were different between OCTAVA and in-built software packages, was tested using Levene’s test for equality of variances, which does not have the constraint of normality. Additionally, a Kruskal–Wallis test was used to test whether the computed OCTA metrics varied across instruments.

### Test–retest repeatability

The repeatability coefficient measures the intrasession repeatability. Two measurements were taken for 30 participants using the RTVue-XR. The OCTAVA test–retest repeatability was measured by calculating the coefficient of repeatability (CR) of OCTA images collected with the RTVue-XR and analysed by OCTAVA. The CR is defined as 2.77 × S_w_ (S_w_ – within standard deviation). We also calculated CR for metrics delivered by the RTVue-XR in-built software. The CR was not calculated for the other instruments since multiple images of the same participant were not available.

## Results

### Study participants

The demographics and ocular biometry of all study participants are summarised in Table 3.

**Table 3:** Participant demographic data and summary of ocular biometry.

Participant parameters	Cirrus 5000, Carl Zeiss Meditec AG	Revo NX 130, Optopol Technology	RTVue-XR Avanti, Optovue Inc	Spectralis OCT2 module, Heidelberg Engineering
Number of participants (n)	32	30	31	27
Number of eyes	32	30	31	27
Age (y), mean (range)	25 (21– 40)	24 (18–30)	33 (23–69)	22 (18–33)
Gender (male:female, n)	16:16	14:16	14:17	12:15
Axial length [mm], mean (SD, range)	25.83 (1.63, 20.43–28.08)	24.56 (1.41, 22.23–28.12	24.32 (1.36, 21.45–27.88)	25.71 (1.63, 23.17–28.44)
Cylinder non-cycloplegic [D], mean (SD, range)	-1.17 (0.65, -2.50 to -0.25)	-0.63 (0.67, -3.87 to +0.5)	-0.53 (0.76, -4.50 to +1.25)	-1.06 (0.97, -3.75 to 0)
Spherical equivalent non-cycloplegic [D], mean (SD, range)	-4.74 (2.97, -10.88 to +1.88)	-2.31 (2.81, -8.11 to +1.98)	-1.70 (2.37, -8.00 to +3.25)	-4.44 (3.1, -11.125 to +0.375)
Corneal curvature [mm], mean (SD, range)	7.70 (0.35, 7.07–8.43)	7.78 (0.29, 7.34–8.41)	7.81 (0.27, 7.19–8.64)	7.92 (0.18, 7.52–8.39)

### Frangi filter grading

There was moderate agreement between all three graders (Fleiss kappa coefficient: 0.421; 95% CI 0.277–0.565). There was good agreement between each grader and the reference standard scores. Fleiss kappa coefficient and precision percentage for graders’ scores versus (vs) the reference standard score were $\kappa$ (precision%) = 0.726 (91%), 0.438 (84%), and 0.850 (96%) for MZ, GRU, and MSD, respectively. Graders MZ vs GRU, MZ vs MSD, GRU vs MSD were, respectively, $\kappa$ = 0.357 (80%), 0.621 (87%), and 0.293 (76%). The graders agreed that a maximum kernel size of 4 pixels for the Frangi filter was optimal. This size was applied to all images as the best trade-off for accurate segmentation of the small and large vessels.

**Figure 2:**
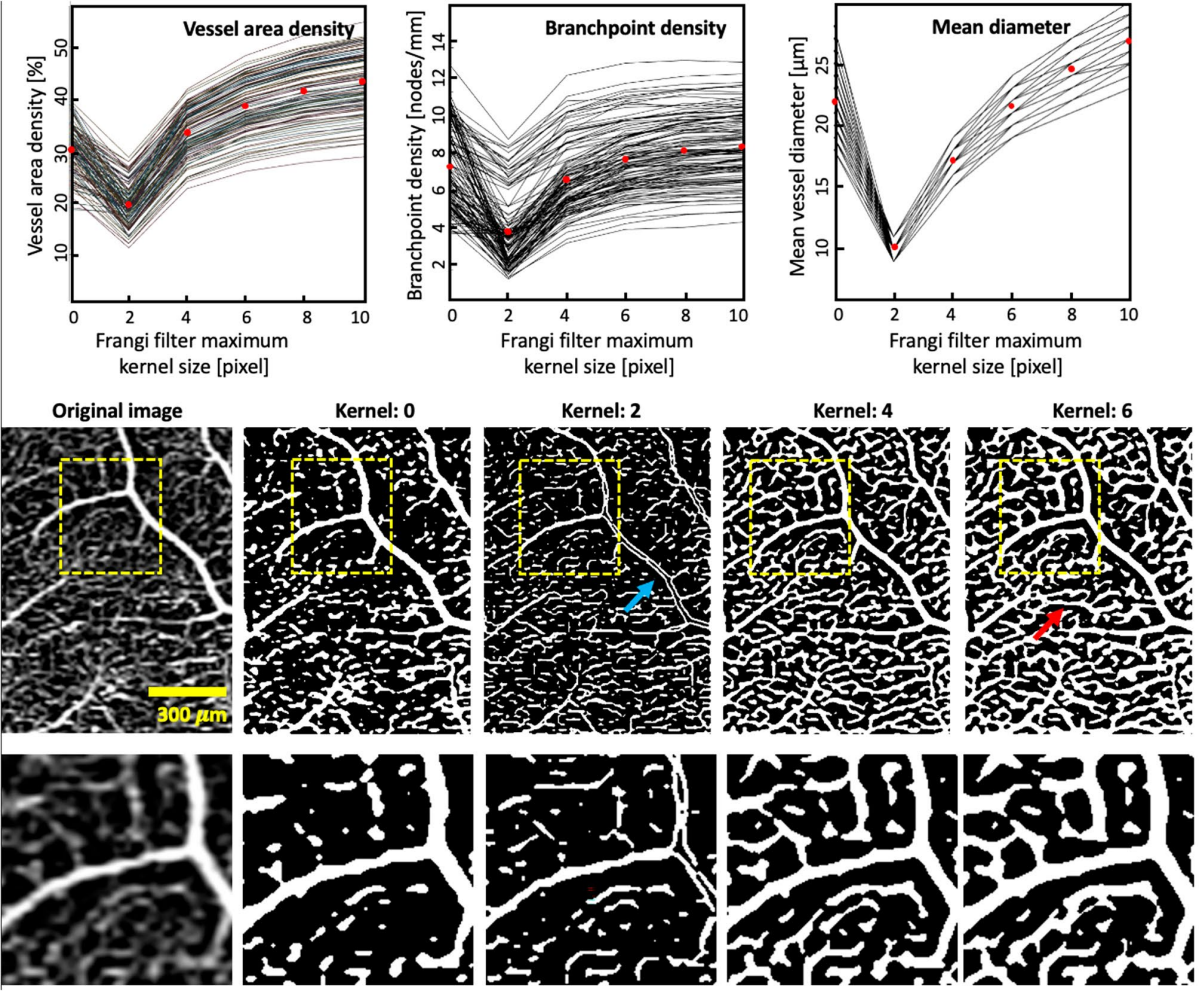
Illustration of how OCTA-derived microvascular metrics (top row) and the optimised image (middle and bottom rows) change with the Frangi filter maximum kernel size. Top row presents metrics from all 120 participants, each represented by one line. Red dots indicate the mean value. The mean diameter plot appears to have fewer lines since many of the lines overlap. Bottom row shows magnified portions highlighted by yellow boxes in the middle row. For a maximum kernel size 2, the filter misidentifies some large vessels, instead representing them as two smaller vessels (blue arrow). With a larger kernel size of 6, some of the smaller vessels appear artificially dilated (red arrow).

Figure 2 and Supplementary Fig. S3 show how selected OCTA metrics change depending on the choice of Frangi filter maximum kernel size and how the kernel size impacts binarization. Overall, small vessels are dilated with increasing size of the kernel and values of most of the metrics increase (but mean tortuosity decreases). The mean VAD [%] of all images for data processed without the Frangi filter is 30.4, and is 19.6, 33.5, 38.7, 41.6, and 43.4, respectively, for maximum kernels of 2, 4, 6, 8, and 10 pixels. For the same maximum kernel sizes, i.e., 0, 2, 4, 6, 8, and 10 pixels, the other metrics vary as follows. The mean total length [mm] (not shown in the graph) is 132, 150, 179, 177, 175, and 173. Mean branchpoint density [nodes/mm] is 6.64, 3.71, 6.06, 6.99, 7.36, and 7.51. The mean diameter [µm] is 22, 10, 17, 21, 25, and 27. Mean tortuosity (not shown in the graph) is 1.14, 1.19, 1.15, 1.12, 1.12, 1.11, and 1.11. The maximum kernel size of 4 pixels represented the first point on the main trend of the graphs for mean VAD, vessel length, mean vessel diameter, mean branchpoint density and the first minimum for mean tortuosity.

### Twig size analysis

Twig size impacts the skeletonization and metrics delivered from skeletonized images, such as vessel total length, branchpoint density, tortuosity and mean and median vessel diameter. The vessel diameter is calculated using a Euclidian distance transform, which measures the perpendicular distance from the vessel centreline (based on the skeletonized image) to the edge (based on the binarized image).

**Figure 3:**
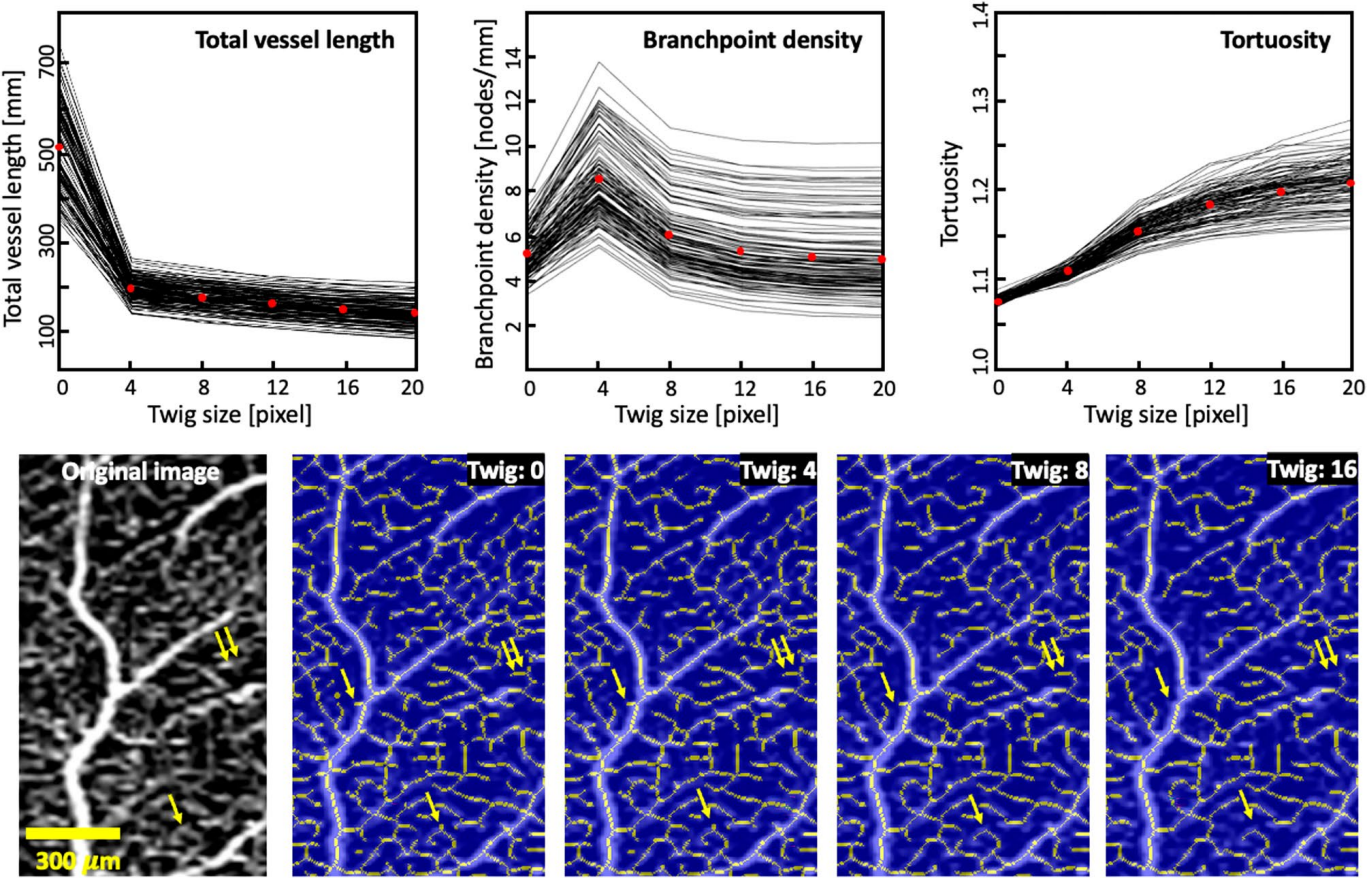
Illustration of how OCTA-derived microvascular metrics (top row) and skeletonized image (bottom row) change with twig size. Top row presents metrics from all 120 participants, each represented by one line. Red dots indicate the mean value. Yellow arrows indicate examples of vessel structures that notably change in the skeletonized image when the twig size is increased.

Figure 3 shows how selected OCTA metrics change depending on the twig size. The mean VAD remains unchanged—as the VAD is calculated based on the binarized image, not the skeletonized image. The mean total vessel length [mm] changes as 518, 197, 177, 161, 149, 139, respectively, for twig size values 0, 4, 8, 12, 16 and 20 pixels. For the same twig size values, the other metrics vary as follows. The mean vessel diameter [µm] is 15, 17, 17, 18, 18, and 18. The mean branchpoint density [nodes/mm] is 5.1, 8.5, 5.9, 5.2, 5.0, and 4.9. The mean tortuosity is 1.07, 1.11, 1.16, 1.18, 1.20, and 1.21. Twig size of 8 pixels is the first point of the main trend of the graphs for branchpoint density and tortuosity.

### OCTAVA settings for retinal OCTA instruments

After carefully examining how various settings impact the retinal OCTAVA-derived metric values, we selected optimised settings for retinal OCTA datasets. All images were up-sampled to 1000 pixels in each direction to ensure that kernel-based filters are applied consistently across all images. There was no median filter applied to any of the images. The fuzzy means algorithm was applied to segment images with the Frangi filter maximum kernel size of 4 pixels. The twig size chosen was 8 pixels. The optimal FAZ segmentation parameters varied by instrument. Once the stringency factor was identified for a particular instrument and the need for tuning was determined, the automated segmentation worked well with the same settings for all images acquired with that instrument. For images acquired with the Cirrus 5000, FAZ segmentation was performed using the Rapid FAZ option with the default stringency factor and no additional active contouring. For images acquired with the RTVue-XR, FAZ segmentation was performed by initialising active contouring with the Rapid FAZ option using a stringency factor at the mid-point of the slider. The Tune FAZ option was used to fine-tune this initialisation. For images acquired with the Spectralis OCT2 module, the Rapid FAZ option was used with a strict stringency factor and no additional tuning with active contouring. For images acquired with the REVO NX 130, FAZ segmentation was performed manually (Draw FAZ function), as the level of noise (due to the combined effects of phase noise and use of the average intensity projection) in the FAZ area prevented accurate segmentation using the automated methods. FAZ segmentation and analysis were done on the SVP layer for all images apart from the RTVue-XR, for which the total retina layer slabs were chosen to enable comparison with in-built RTVue-XR software. The assessment of optimal parameters for FAZ segmentation was done by visual inspection (DMS).

### Comparison of OCTAVA with in-built OCTA software

Table 4 summarises the quantitative comparison of the OCTAVA metrics with their counterparts in the commercial in-built software packages available from three of the four instruments. Overall, OCTAVA generates similar or highly correlated values to Cirrus 5000 for almost all metrics. The Spearman correlation coefficient suggests a strong significant positive correlation for 8 of the 9 OCTA metrics we evaluated, while a Bland–Altman analysis indicated no significant differences in the ranges of values for 5 of the 9 metrics. This implies that the metrics may be computed similarly by OCTAVA and the Cirrus 5000 in-built software. There were significant differences between all metrics values generated by OCTAVA and the Revo NX 130 software. A strong and positive Spearman correlation was observed only for the metrics FAZA and FAZ perimeter, however, the Bland-Altmann analysis indicated a significant difference in the range of values (p < 0.01) for all metrics. A strong and positive correlation between all microvascular metrics delivered by OCTAVA and RTVue-XR is also seen. However, Bland–Altman analysis shows that only the outcome of FAZ analysis is similar between the software packages. The values of vessel area density are different between all software packages, with OCTAVA providing similar values to the Cirrus 5000, but much lower values than the RTVue-XR and Revo NX 130.

**Table 4:** Bland–Altman analysis and paired t-tests show the level of agreement between OCTAVA and in-built commercial OCTA software packages for measuring retinal vessel area density, vessel length, and foveal avascular zone metrics.

Instrument and participant count	Metric	OCTAVA	In-built software	Bland–Altman analysis						Spearman correlation (p-value)
		Mean (SD)	Mean (SD)	Mean	SD	Lower LA	Upper LA	t_stat_	p-value	
Cirrus 5000 n = 32	Total VAD [%]	36.5 (1.9)	35.6 (2.1)	+0.9	1.72	-2.4	+4.3	+3.1	< 0.01	0.630 (p < 0.01)
	Parafoveal VAD [%]	37.4 (2.2)	37.9 (2.1)	-0.5	1.77	-3.9	+3.0	-1.5	< 0.01	0.667 (p < 0.01)
	Foveal VAD [%]	19.1 (4.2)	17.5 (4.5)	+1.6	0.17	+0.1	+1.0	+21.6	< 0.01	0.905 (p < 0.01)
	Total VL [mm]	185 (11)	183 (10)	+2	7.5	-12.8	+16.5	+1.4	0.17	0.724 (p < 0.01)
	Parafoveal VL [mm]	132.5 (8.6)	152.3 (7.9)	-19.8	5.5	-30.5	-8.9	-20.3	< 0.01	0.792 (p < 0.01)
	Foveal VL [mm]	7.7 (5.6)	8.4 (2.1)	-0.7	5.5	-11.4	+10.0	-0.7	0.48	0.388 (p = 0.28)
	FAZA [mm^2^]	0.275 (0.107)	0.275 (0.11)	-0.001	0.019	-0.04	+0.04	-2.7	0.79	0.923 (p < 0.01)
	FAZ perimeter [mm]	2.29 (0.52)	2.17 (0.43)	+0.11	0.28	-0.43	+0.66	+2.3	0.03	0.799 (p < 0.01)
	FAZ circularity	0.67 (0.15)	0.71 (0.07)	-0.04	0.11	-0.25	+0.16	-2.3	0.03	0.708 (p < 0.01)
Revo NX 130 n = 30	Total VAD [%]	31.9 (3.7)	40.3 (1.2)	-8.3	3.4	-15.4	-1.3	-12.7	< 0.01	0.192 (p = 0.31)
	Total VL [mm]	178 (20)	208 (11)	-30	19	-68	+6	-8.9	< 0.01	0.406 (p = 0.03)
	FAZA [mm^2^]	0.322 (0.144)	0.342 (0.163)	-0.02	0.04	-0.091	+0.051	-3.0	< 0.01	0.955 (p < 0.01)
	FAZ perimeter [mm]	2.28 (0.59)	2.48 (0.69)	-0.20	0.38	-0.95	+0.55	-2.9	< 0.01	0.843 (p < 0.01)
	FAZ circularity	0.77 (0.17)	0.58 (0.097)	+0.20	0.19	-0.17	+0.56	+5.75	< 0.01	0.176 (p = 0.35)
RTVue-XR n = 31	Total VAD [%]	30 (3)	50 (2)	-20	1.7	-23.7	-17.1	-43.6	< 0.01	0.705 (p < 0.01)
	FAZA [mm^2^]	0.213 (0.111)	0.212 (0.11)	+0.001	0.015	-0.027	+0.030	+0.5	0.63	0.992 (p < 0.01)
	FAZ perimeter [mm]	2.09 (0.7)	1.77 (0.49)	+0.32	0.37	-0.40	+1.04	+4.8	< 0.01	0.884 (p < 0.01)
	FD-300 [%]	34.3 (4)	50 (4)	-15.6	2.1	-19.7	-116	-42.2	< 0.01	0.783 (p < 0.01)

OCTAVA metrics were generated with Frangi filter maximum kernel size 4 pixels and twig size 8 pixels.

*VAD* vessel area density, *VL* vessel length, *FAZA* foveal avascular zone area.

Supplementary Table S2 shows the histogram parameters for selected microvascular OCTA metrics and FAZ metrics for 93 images captured by the Cirrus 5000, Revo NX 130 and RTVue-XR instruments. Overall, when all data is analysed together, a reduced variation in vessel area density is seen for OCTA images processed by OCTAVA compared to values aggregated from the three software packages. The variance of values generated by OCTAVA vs the aggregated commercial software packages is 33% vs 42%. Levene’s test reveals a difference in variance in all VAD metrics when comparing OCTAVA to in-built software packages on the same images (Supplementary Table S3, Supplementary Fig. S4). Similarly, a Kolmogorov–Smirnov (KS) test reveals a difference in the distribution of VAD and total length metrics as computed by OCTAVA versus in-built software. There is no difference in data distribution for FAZ analysis (KS test: p = 0.96 and p = 0.24 for FAZ area and perimeter, respectively). Interestingly, when comparing across instruments, OCTAVA generally shows more consistency in computing FAZ perimeter (Kruskal–Wallis, p = 0.65) and total length (p = 0.18). In-built software packages show a drastic difference in the means of these two metrics across instruments (p < 0.01 for both) (Supplementary Table S3, Supplementary Fig. S4). Both OCTAVA and in-built software show differences across instruments for the VAD and FAZ area metrics.

### Coefficient of repeatability

Coefficient of repeatability (CR) values for metrics generated by OCTAVA are similar to the CR values of metrics generated by in-built RTVue-XR software. For OCTAVA vs in-built RTVue-XR software, respectively, FAZ CRs are: 0.028 vs 0.024 [mm^2^], FAZ perimeter CRs are: 0.499 vs 0.417 [mm], FD-300 CRs are 4.3 vs 3.5 [%], total retinal VAD CRs are: 3.2 vs 2.9 [%], foveal VAD CRs are: 2.4 vs 3.0. The CR values provided by OCTAVA are comparable to those reported in the literature^43^.

## Discussion

### Need for multiple OCTA-derived microvascular metrics

The retinal microvascular network forms a complex architecture of interconnected vessels^44^. Such complexity strongly suggests that full characterisation of changes in vessel morphology will require multiple metrics^45,46^. In addition, since many diseases affect multiple markers, it is important in building a complete picture of a condition to use multiple metrics, even if ultimately a minimum set of biomarkers is desirable. This rationale is behind our choice of seven metrics to comprehensively characterise the microvascular network architecture and six to characterise the FAZ. Our choices are based on an extensive qualitative review of the literature, balancing coverage and overlap of characteristics.

### Optimal OCTAVA settings

As expected,^47^ changing parameters within OCTAVA, such as the Frangi filter maximum kernel size and twig size, significantly alters quantitative measurements thereby motivating our rigorous analysis to establish optimal parameters. We concluded, for the instruments used in our study and the binarization method used in OCTAVA, that the optimal Frangi filter maximum kernel size is 4 pixels and the optimal twig size is 8 pixels. It is important to note that to properly compare images from different systems using OCTAVA, the pixel density must be the same, as parameters defined in the analytical pipeline are specified in units of pixels. In our study, all images were rescaled to 1000 $\times$ 1000 pixels. We suggest that, for consistency and for the purpose of comparison, others resample their images similarly or at least report the pixel density and OCTAVA settings they are using. We anticipate that these parameter values are a useful starting point for other applications of OCTAVA to retinal OCTA, whilst recognising that further work to develop better filtering and binarization methods would be helpful and may impact on the optimal values.

### OCTAVA microvascular metrics vs in-built software

As expected, the OCTA-derived metrics generated by OCTAVA and in-built software packages are similar for some instruments but not for others. OCTAVA generates similar values to the Cirrus 5000 for 5 of the 9 metrics we assessed but not to the other commercial software packages tested here, although a strong and positive correlation can be seen for selected metrics.

Differences in OCTA-derived microvascular metrics values caused by the algorithm used for analysis, as well as other factors, have been reported in the literature before. In their review paper, Hormel and Jia summarised that differences in vessel area density values made on the same eye can be due to scan location, pattern and size, thresholding, and filtering methods^48^. Sampson et al. reported reductions in vessel area density due to an RTVue-XR in-built software upgrade^49^. Rabiolo et al. applied seven different segmentation algorithms to one OCTA dataset^50^. At the end of the binarization process, the ratio between the number of white pixels and the number of total pixels (VAD) was calculated for each image and differed by up to 40% between methods (repeated measures ANOVA, p < 0.0001). Corvi et al. evaluated the reproducibility of parafoveal microvascular anatomy of seven different OCTA devices by comparing VAD, fractal dimension and FAZA of the superficial vascular plexus in the same study cohort^51^. Despite analysing images using the same analysis pipeline, the authors showed significant variability in metrics (Friedman test, p < 0.0001). However, it is difficult to interpret their results since the segmentation method used is not fully specified and other factors may have contributed. For example, the authors indicate use of a global thresholding algorithm applied in ImageJ, which did not always perform well on OCTA images in our experience^17^. The differences they observed could also be caused by different segmentation boundaries, number sampling density, or flow detection range used by each instrument. Additionally, as seen in the first figure of the article, and pointed out by Sacconi et al., images from different devices may not be properly overlapped before analysis, which would also impact the values of metrics obtained^52^. Magrath et al. evaluated the FAZA and VAD variability between the RTVue-XR and Cirrus 5000^53^. They compared results from the RTVue-XR in-built software with manual analysis in ImageJ for data from both OCTA instruments. For the same cohort, they found statistically similar values between the RTVue-XR in-built software and manually analysed images from the Cirrus 5000, but significantly different values compared to manual analysis of images from the RTVue-XR (Student’s t-test, p = 0.0396 and p =  < 0.0001 for FAZA and VAD, respectively). Munk et al. imaged nineteen study participants using four commercial devices: Cirrus 5000, RTVue-XR, Spectralis OCT2 module, and DRI OCT Triton (Topcon Healthcare, Tokyo, Japan)^54^. Image analysis was undertaken with the Angiotool software^10^. The authors reported that there was no significant difference in overall VAD among the instruments used when analysed using the same software (48.7, 47.9, 48.3, and 46.5%, respectively). The results of Munk et al. support our proposition that the large variability of metrics extracted from images with different instruments can be mitigated by using a standardised image processing protocol. Our study showed that OCTAVA applied to datasets obtained by different instruments reduces differences in mean values and overall variation between metrics obtained from data sets obtained from different instruments and different healthy study participant cohorts. VAD, total length, and FAZ perimeter had lower variance across instruments when OCTAVA was used for OCTA image processing and analysis (Kruskal–Wallis test, p = 0.40, 0.18, and 0.65 for VAD, total length, and FAZ perimeter, respectively) (Supplementary Tables S2 and S3, Supplementary Fig. S4), supporting that using OCTAVA for analysis is more consistent than using in-built software.

For several of the metrics we assess in this study, including mean vessel diameter, branchpoint density, tortuosity, and fractal dimension, no equivalent metrics were available in the in-built software from any of the instruments. Previously, we assessed the accuracy of these metrics compared with manual characterisation using simulated OCTA data^17^. We have further demonstrated that these metrics can enable discrimination between disease and control groups in cutaneous OCTA images^18^. Whereas we do not yet directly assess the relevance of these metrics for differentiating between disease and control groups for retinal OCTA images, other studies have indicated that these metrics have value^3^. This is a topic for future study.

### OCTAVA microvascular metrics vs histology

As just discussed, the retinal OCTA literature reports a wide range of values for microvascular metrics^55^. Arguably, histology is the gold standard for tissue analysis more generally. Indeed, for microvasculature, histology currently suffers from fewer artefacts than OCTA and enables visualisation with a better signal-to-noise ratio. Thus, we compared OCTAVA-generated microvascular metrics values with histology in several studies in which retinal ex vivo images were analysed and vessel area density and branchpoint density were reported. Mendis et al. reported mean VAD for retinal images of human donor eyes imaged with a confocal scanning laser microscope: mean SVP VAD in the temporal and inferior regions, respectively, (1.5 mm from the fovea, area: 1.3 × 1.3 mm) was 41.0 and 41.2%^56^. Yu et al. studied macular microvasculature metrics using human donor eyes and confocal imaging^57^. They reported mean vessel density for all vessel plexuses above the deep layer to be 31, 34, and 29% for the total retina, parafoveal, and foveal regions, respectively. Yu et al. also measured VAD in the temporal and inferior regions to be 30 and 33%, respectively. The VAD values generated by OCTAVA fall within the range of those obtained by confocal microscopy of ex vivo tissue (with mean VAD for all 120 images of 33%). We note that VAD values generated by the RTVue-XR in-built software are notably larger than those obtained using confocal microscopy (mean VAD of 50%).

Branchpoint density for the four instruments measured using OCTAVA is 5.5, 5.1, 4.7, and 7.8 nodes/mm, respectively, for the Cirrus 5000, Revo NX 130, RTVue-XR, and Spectralis OCT2 module. We identified one report based on histology in which the branchpoint density was reported as between 5.4 and 7.4 nodes/mm depending on the location^55^.

### Limitations and future directions

Our study confirms the observation made by other researchers that values generated for OCTA-derived microvascular metrics depend on the image analysis workflow. A standardised set of well-defined metrics, and standardised and widely applied image analysis pipeline are needed to minimise systematic differences in metrics caused by differences in definitions and image analysis. OCTAVA contributes to standardising the workflow by allowing access to images generated at different stages of the analysis for evaluation in an easily accessible format to maximise transparency and shareability between users. Notably, we did not have access to the binarized and skeletonized images generated by commercial software. Access to these intermediate steps in the pipeline would enable us to better understand the differences we see between OCTAVA-derived and commercial in-built software-derived microvascular metrics. This lack of transparency underscores the need for open-access software for analysing OCTA images to improve image analysis and, ultimately, through better defined and more sensitive disease biomarkers, improve patient care.

One notable limitation of any quantitative analysis beyond the in-built software is the variable pixel density of images exported by each instrument. Although each of the instruments we tested has comparable resolution, the pixel density of the exported images is highly variable between instruments. We note the importance of resizing all images to the same size for a consistent analysis, which enables the application of image processing steps and filter kernels uniformly across the whole dataset. However, image rescaling cannot recover information lost due to reduced pixel density. This will introduce some inherent bias in the results and particularly impacts metrics which rely on the ability to resolve small features such as mean length and branchpoint density.

OCTAVA processes MIPS and so does not intrinsically deal with projection artefacts. All data considered here were generated for the superficial vascular plexus, thus, avoiding this very important issue. In future, OCTAVA could include software for generating MIPs free from projection artefacts. In the interim, users analysing deeper vascular plexuses should be aware that the presence of projection artefacts will confound the metrics. This is an important consideration regardless of whether OCTAVA or in-built software is used.

OCTAVA does not yet have an in-built function to automatically control image quality and exclude images of poor quality. The OCTAVA user must assess image quality prior to the analysis and exclude those of poor quality. We recommend the following papers discussing criteria for quality assessment in retinal and choroidal OCTA^22,23,58^.

Further, we note that the current version of OCTAVA has been optimised for macular OCTA only, and not for images of the optic disc region. OCTA images of the optic disc could provide additional diagnostic information for some common diseases including glaucoma and other optic neuropathies^59^. However, such images are characterised by specific features including large peripapillary vessels, which may require further image processing and optimisation of filter parameters in order to obtain accurate segmentation. Optimisation of OCTAVA for optic disc images is a topic for further research.

Still better filtering could lead to a more accurate representation of the vessel network in the binarized image. As seen in the images delivered by OCTAVA, some small vessels appear wider than in the original image due to artificial dilation caused by the Frangi filter. Additionally, some of the larger vessels can be missed in the segmentation if the maximum Frangi filter size is too small. Such artefacts are exacerbated by the large size distribution of vessels in the retina, which makes it challenging to select a maximum kernel size which accurately identifies the larger vessels without dilating the smaller vessels. This could be addressed by first segmenting the image to separate the large and small vessels, applying the Frangi filter to each separately, and then merging the filtered images^13,60^. We note that investigation of new vesselness filters and their optimisation for OCTA is an active area of research^61^.

Machine learning methods have demonstrated success in improving the accuracy of segmentation in other medical and non-medical image processing tasks. Such methods would likely improve on some aspects of binarization within OCTAVA, such as ignoring noise within the FAZ (especially an issue in the REVO NX 130 images). However, many of these methods are supervised learning methods, and the acquisition of accurate ground truth for training the machine learning algorithms relies on manual annotation of large datasets, which is difficult, time-consuming, and often unreliable^13,62^. Sophisticated training and data management schemes would be necessary to implement such methods. So far, we have opted not to implement machine learning-based segmentation in OCTAVA to avoid these challenges and to enable use on a broad range of imaging sites beyond the retina.

Our pilot data demonstrate similarity in the values for metrics obtained by OCTAVA and histology, as well as reduced variation in OCTA-derived metrics between commercial instruments. The variance of values generated by OCTAVA is 33% vs 42% for the commercial software packages. Nonetheless, further work is required to fully explore the potential of retinal OCTAVA as a reliable and reproducible OCTA image analysis toolbox. One limitation of the current study is that it is retrospective, with data collected at three different sites on different study cohorts. Ideally, a larger study in which all instruments image the same cohort is required to better validate OCTAVA and identify more subtle improvements. Such further validation could also use a wider range of OCTA instruments and consider a more diverse study cohort, including participants with retinal diseases. This extension is planned for in the next round of OCTAVA improvement and validation.

## Conclusion

We introduced an open-source, robust, easy-to-use retinal extension to the OCTAVA toolbox that provides seven microvascular and six foveal avascular zone metrics. We validated OCTAVA using images collected by four commercial OCTA instruments demonstrating robust performance across datasets from different instruments acquired on different study cohorts at different sites. We discussed the impact of various parameters on metrics and provided a set of parameters for achieving accurate segmentation and measurements of microvascular and FAZ metrics. We showed that OCTAVA delivers retinal microvascular metrics comparable to the literature and reduces their variation between studies compared with in-built software. We highlighted the importance of pixel density when comparing quantitative results from different instruments. OCTAVA is publicly available to expand standardised research and thereby improve the reproducibility of quantitative analysis of retinal microvascular imaging. Further research should focus on validating and optimising OCTAVA on images collected by a still wider range of commercial instruments, all measured on the same but larger and more diverse subject cohort, including healthy and diseased eyes. Widespread use of a standardised toolbox such as OCTAVA will minimise the systematic variability in OCTA-derived metrics currently due to the differences in image analysis software used by different study sites. Ultimately, this standardisation will help to generate more reliable microvascular metrics and build the unified, multi-site large datasets necessary to identify the most general and sensitive biomarkers of retinal and systemic health conditions.

### Supplementary Information

Supplementary Information.

## Data Availability

The datasets generated during and/or analysed during the current study are available from the corresponding author upon reasonable request.
