# Supplementary material for: Towards standardising retinal OCT angiography image analysis with open-source toolbox OCTAVA
Source: Sci Rep. 2024 Mar 12;14:5979. doi: 10.1038/s41598-024-53501-6 (PMC10933365; doi:10.1038/s41598-024-53501-6)
Supplement: Supplementary file 1 — Supplementary Information. [file 41598_2024_53501_MOESM1_ESM.docx]

**Towards standardising retinal OCT-angiography image analysis with open-source toolbox OCTAVA**

# Supplemental Tables and Figures


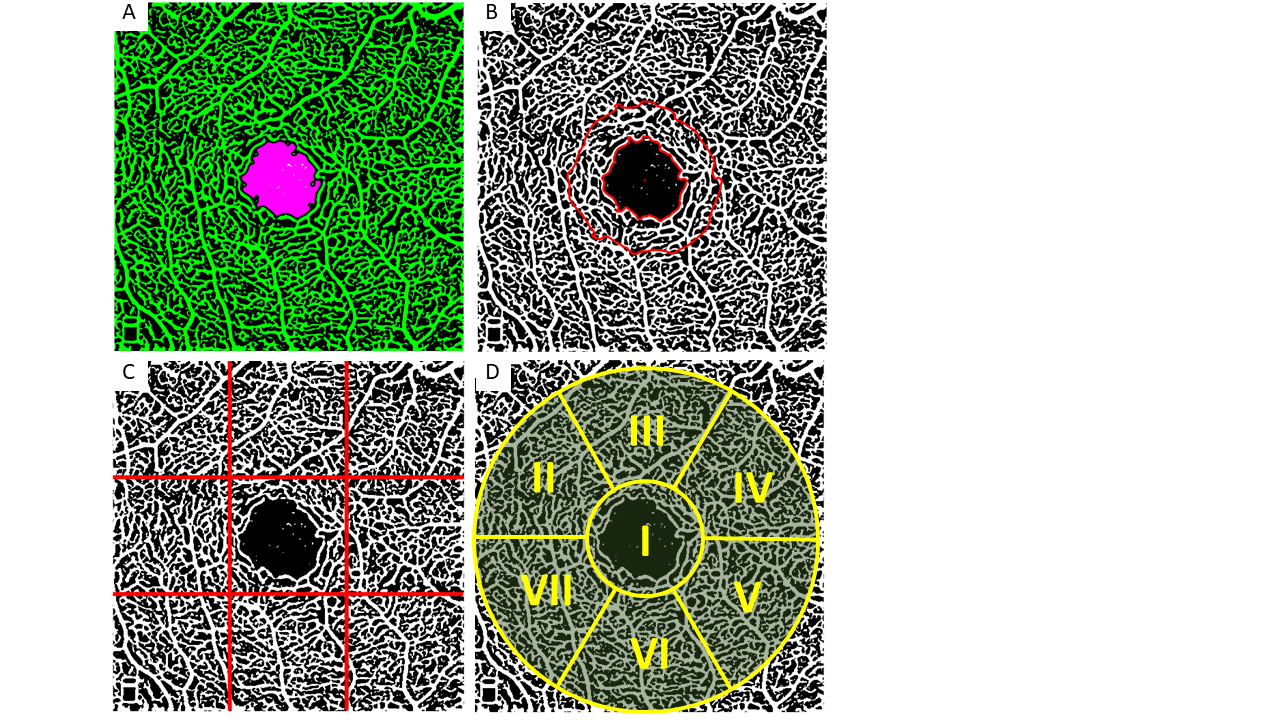


**Supplementary Figure 1**. Additional metrics specific to retinal analysis. FAZ is segmented using pre-initialisation of the contour (A) (pink region) and is optimised using an active contour. The final contour is shown in the inner ring in (B). The outer ring indicates an area within 300 microns of the FAZ contour required for the FD-300 metric. Regional analysis can be performed on a user-defined grid (not shown), on a grid of nine squares (C) or on eight subregions defined by the Early Treatment Diabetic Retinopathy Study grid (D), which includes: foveal circle (I); parafoveal rim (II-VII); superior hemisphere (II-IV); inferior hemisphere (V-VII); temporal region (II and VII); superior region (III); nasal region (IV and V); and inferior region (VI).


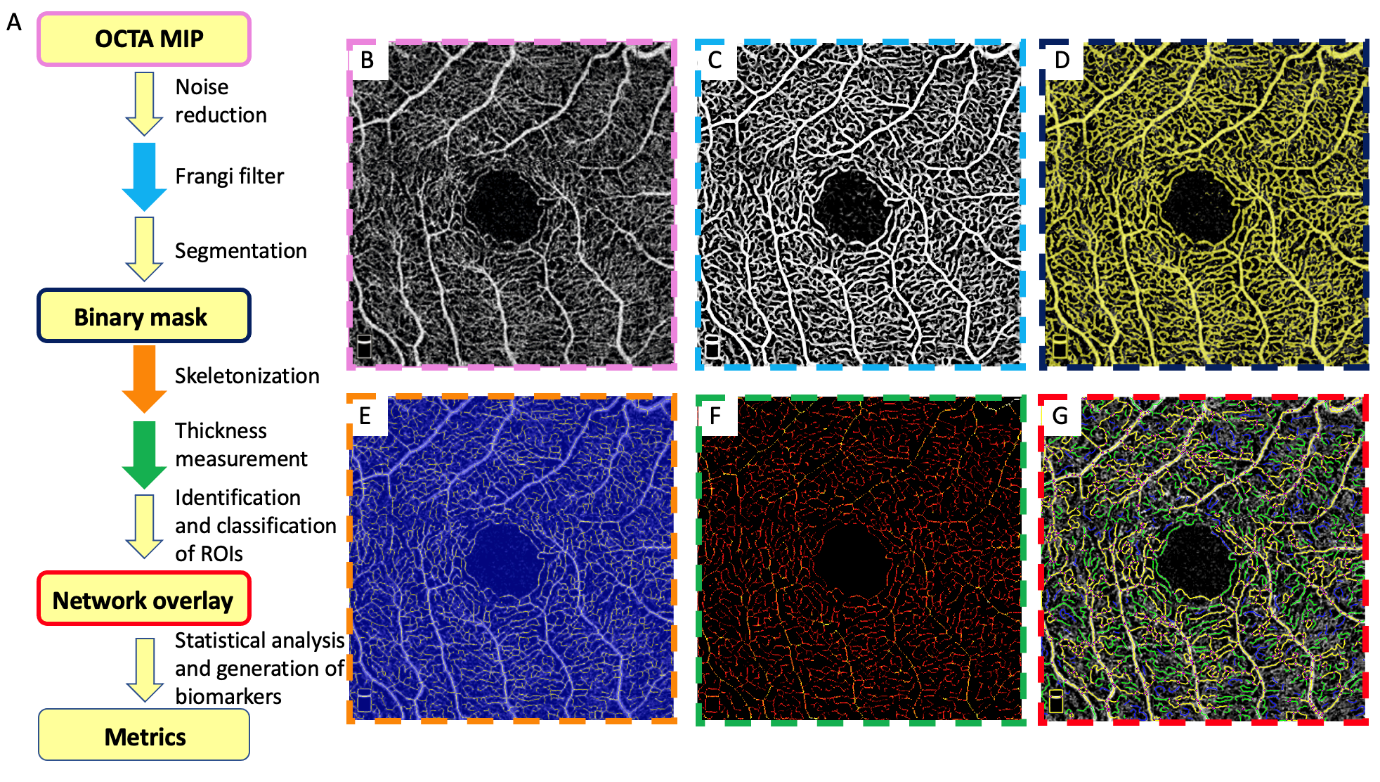


**Supplementary Figure 2**. Flowchart of software workflow (A) and example images generated at each step (B-G). Coloured borders in B-G correspond to the step with the matching-coloured arrow or box in (A). OCTA MIP image (B) is loaded into OCTAVA. Images are filtered using an optional median filter to reduce noise and Frangi filter to enhance the intensity of vessel-like structures (C). Images are segmented using the chosen method to create a binary mask, which is displayed overlaid on the original MIP image so the user can assess the accuracy of the segmentation (D). The network is skeletonized, and the skeletonized image is displayed overlaid on the original MIP image (E). The thickness of the vessels is measured using a Euclidian distance transform (F). Finally, nodes and endpoints are identified, and vessels are categorized by their interconnectivity to the network. The network is displayed overlaid on the original MIP image (G). Colours in G represent connectivity to the network: segments (yellow) are connected to nodes on both ends, branches (green) are connected to one node and one endpoint, and isolated elements (blue) are connected to two endpoints.

| **Metric (abbreviation)** | **Unit** | **Analysed image** | **Description and importance** |
| --- | --- | --- | --- |
| Vessel area density (VAD) | % | Binarized | Perfused blood vessel area (white pixels) in the binarized OCTA MIP image divided by total image area (all pixels) multiplied by 100. Can be used to demonstrate ischemia. When calculated in a user-defined sub-region, can be used to calculate flow area, e.g., for assessing flow in the vessel-free region between the outer plexiform layer and Bruch’s membrane. |
| Vessel length density (VLD) | % | Skeletonized | Total vessel length multiplied by the width of one pixel (i.e., total area of skeletonized vessels) divided by the total image area and multiplied by 100. Length measurements are calculated from the skeletonized OCTA MIP image. More sensitive than VAD to perfusion changes at the capillary level. |
| Mean, median, and distribution of vessel diameter | μm | Binarized | Mean, median, and distribution of diameter measurements are acquired using a Euclidian distance transform applied to the binarized OCTA MIP. The mean (median) diameter is the mean (median) for a single vessel, including one value for each identified segment. Can inform on dilation, sprouting, or vessel regression. |
| Total, mean, median, and distribution of vessel length (VL) | μm | Skeletonized | Total, mean, median, and distribution of the single-vessel centreline lengths including one value for each identified segment measured from the skeletonized image. Network interconnectivity and branching patterns can indicate oxygenation/nutrient delivery dysfunction. |
| Branchpoint density (BD) | nodes/mm | Skeletonized | Number of identified nodes divided by total vessel length. May indicate resilience to occlusion or blockage of blood flow. |
| Mean (MeanT) and distribution of vessel tortuosity | 1 | Skeletonized | Tortuosity is calculated from the arc length-over-chord ratio. Can inform on pathological microvascular remodelling and/or ischemia. |
| Fractal dimension (FD) | 1 | Binarized | Indication of how the network fills space on variable length scales, calculated using the box counting method. FD is commonly reported as FD ± FD_error_. Altered spatial distribution of the capillary network is an indicator of impairment of oxygenation and nutrient delivery. |
| Foveal avascular zone area (FAZA) | mm^2^ | Binarized | Total area of black pixels enclosed by the FAZ segmentation contour. Changes in the FAZA can be indicative of macular oedema or ischemia and changes in retinal thickness. |
| FAZ perimeter | mm | Binarized | Length of the perimeter of the FAZ segmentation contour. It provides an additional metric about the change in FAZA. |
| FAZ axis ratio | 1 | Binarized | Ratio between the major and minor axis of the ellipsoidal best fit to FAZ outline shape. Elongation of the FAZA can be indicative of changes in retinal thickness. |
| FAZ circularity | 1 | Binarized | FAZ area multiplied by 4π divided by FAZ perimeter. Indicates elongation of the FAZ, which can be indicative of changes in retinal thickness. |
| FAZ acircularity index | 1 | Binarized | Ratio between the measured perimeter and the perimeter of a circular  area of the same size. Indication of elongation of the FAZ. |
| Foveal avascular density 300 (FD-300) | % | Binarized | Percentage of the area occupied by vessels (white pixels) divided by the area in a 300-μm width rim surrounding the FAZ. This rim width value was originally chosen based on the relationship between FAZ and ganglion cell complex thickness in normal eyes to better distinguish between normal variations in FAZ and those due to pathology. |

**Supplementary Table 1.** Summary of the metrics generated by OCTAVA. A unit of 1 indicates dimensionless.


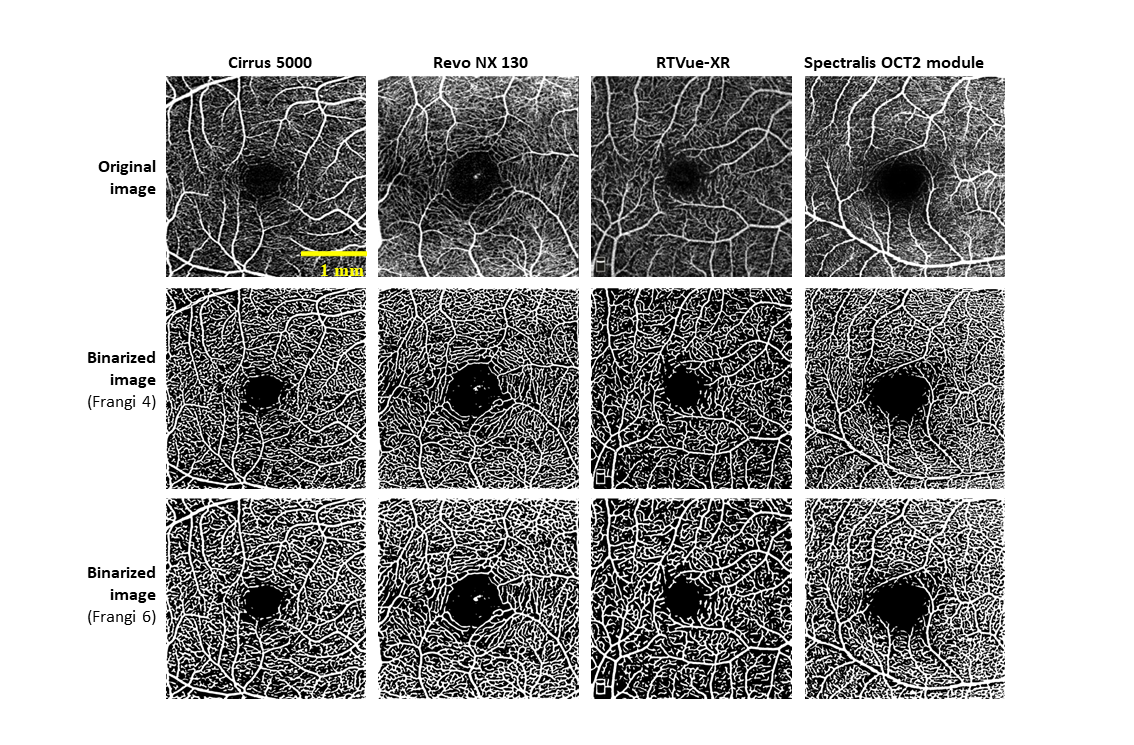


**Supplementary Figure 3.** Illustration of how binarized images (top row) change with the Frangi filter maximum kernel size 4 (middle row) and 6 (bottom row) for each instrument used in the study imaging a different subject.

|  | **Total VAD [%]** | | **Parafoveal VAD [%]** | | **Foveal VAD [%]** | | **FAZ area [mm^2^]** | | **FAZ perimeter [mm]** | |
| --- | --- | --- | --- | --- | --- | --- | --- | --- | --- | --- |
|  | **OCTAVA** | **Commercial** | **OCTAVA** | **Commercial** | **OCTAVA** | **Commercial** | **OCTAVA** | **Commercial** | **OCTAVA** | **Commercial** |
| Number of images | 93 | 93 | 63 | 63 | 63 | 63 | 93 | 93 | 93 | 93 |
| Mean | 33 | 42 | 34 | 45 | 19 | 22 | 0.268 | 0.276 | 2.21 | 2.14 |
| Variance | 16 | 39 | 19 | 57 | 17 | 48 | 0.016 | 0.019 | 0.378 | 0.373 |
| Skewness | -0.34 | +0.39 | -0.31 | +0.07 | -0.69 | +0.04 | +0.52 | +0.67 | -0.38 | +0.44 |
| Kurtosis | -0.43 | -1.14 | -0.36 | -1.72 | +0.89 | -0.25 | +0.30 | +0.04 | +0.61 | +0.26 |
| Range | 19 | 22 | 19 | 22 | 21 | 31 | 0.66 | 0.64 | 3.26 | 3.13 |
| Minimum | 23 | 32 | 22 | 35 | 5 | 5 | 0.01 | 0.04 | 0.43 | 0.79 |
| Maximum | 42 | 54 | 41 | 57 | 26 | 36 | 0.67 | 0.68 | 3.69 | 3.92 |

**Supplementary Table 2.** Histogram analysis of selected OCTA microvascular metrics obtained from three instruments, Cirrus 5000 (number of images, n=32), Revo NX 130 (n=30) and RTVue-XR (n=31) analysed by in-built software and OCTAVA.

| **Test** | **Total VAD [%]** | **Parafoveal**  **VAD [%]** | **Foveal VAD [%]** | **Total length [mm]** | **FAZ area [mm^2^]** | **FAZ perimeter [mm]** |
| --- | --- | --- | --- | --- | --- | --- |
| KS test p-value (commercial vs OCTAVA), Figs. A-D | <0.01 | <0.01 | <0.01 | <0.01 | 0.96 | 0.24 |
| Levene’s test p-value (commercial vs OCTAVA), Figs. A-D | <0.01 | <0.01 | <0.01 | 0.41 | 0.40 | 0.88 |
| Kruskal-Wallis test p-value | | | | | | |
| All instruments – commercial software analysis, Figs. E-H | <0.01 | <0.01 | <0.01 | <0.01 | <0.01 | <0.01 |
| All instruments – OCTAVA analysis, Figs. I-L | <0.01 | <0.01 | 0.40 | 0.18 | 0.011 | 0.65 |

**Supplementary Table 3.** P-values from statistical comparisons of metrics generated by OCTAVA and in-built software. The Kolmogorov-Smirnov test was used to detect differences in distributions of metrics computed by OCTAVA and in-built software on the same images. Similarly, Levene’s test was used to determine whether the variances of metrics computed by OCTAVA and in-built software were different. The Kruskal-Wallis test was used to detect differences in the sample means of metrics from images collected on different instruments. For this analysis, OCTAVA metrics and in-built metrics were compared separately. Figs. A – L are in Supplementary Figure 4.


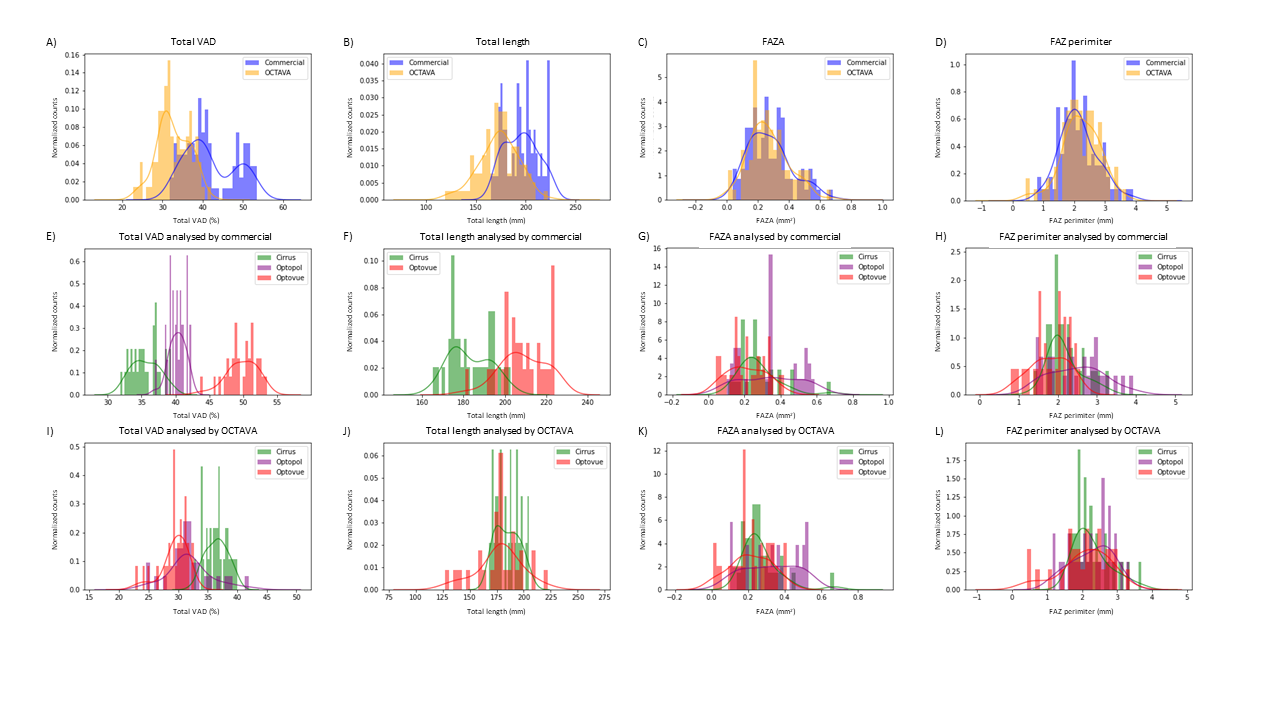


**Supplementary Figure 4.** Histograms of select metrics compared in Supplementary Table 3. A-D: Total VAD (A), Total VLD (B), FAZ area (C), and FAZ perimeter (D) computed by OCTAVA (orange) versus in-built software from commercial systems (blue). E-H: Metrics computed by commercial in-built packages are compared across collection instruments. I-L: Metrics computed by OCTAVA are compared across collection instruments. Brown colour in A-D and dark red in E-L indicate overlap of the histograms.
